# Hyperacusis in gifted children and its impact on quality of life

**DOI:** 10.1007/s00405-025-09389-7

**Published:** 2025-04-12

**Authors:** Banu Baş, Rukiye Çolak Sivri

**Affiliations:** 1 Faculty of Health Sciences, Department of Audiology, Ankara Yıldırım Beyazıt University, Esenboğa Külliyesi Dumlupınar Mahallesi Küme Evleri Çubuk, Ankara, 06760 Turkey; 2 Rukiye Çolak Sivri Speciality Clinic, Ankara, Turkey

**Keywords:** Hyperacusis, Gifted children, Quality of life

## Abstract

**Purpose:**

The aim of this study is to determine the presence of hyperacusis in gifted children and evaluate its effect on quality of life.

**Methods:**

The study included 47 children between the ages of 7 and 12 who did not have hearing loss, who were diagnosed as gifted by scoring 120 or more on the Weschler Intelligence Scale for Children-IV (WISC-IV) and who did not have any accompanying disabilities, and 27 age and gender matched children with normal intelligence.The ‘Pediatric Hyperacusis Questionnaire Parent Form(P-HQ)’ was applied to the children for hyperacusis assessment, and the ‘Quality of Life Inventory for Children (PedsQL)’ was applied for quality of life.

**Results:**

When the pediatric hyperacusis questionnaire parent form scores were compared between the groups, a statistically significant difference was obtained (*p* =.000).In the quality of life assessment made using the quality of life scale for children, a statistically significant difference was obtained between the groups in the sub-parameters of the test; physical health total score (*p* =.000),emotional functioning score (*p* =.032),social functioning score (*p* =.007),school functioning score (*p* =.000) and scale total score (*p* =.000).

**Conclusion:**

Our study results showed that gifted children mostly complain of hyperacusis and that this complaint increases as their intelligence level increases.We believe that hyperacusis and its effect on quality of life should not be ignored in gifted children.Whenever possible, gifted children should be evaluated for hyperacusis and sensory sensitivities.We think that individualized therapy planning is necessary for the difficulties caused by sensory sensitivities that affect quality of life. Our findings revealed the necessity of hyperacusis assessment and therapy when working with gifted individuals.

## Introduction

Giftedness refers to an individual’s exceptional cognitive, creative, or physical abilities in a specific domain, often demonstrated through performance surpassing the expectations for their age. It represents overall cognitive capacity, with such children excelling in cognitive skills like rapid learning, abstract reasoning, and problem-solving [1]. Gifted children are characterized not only by their cognitive capacity but also by their creative thinking, specialized talents, and extraordinary skills in particular fields [2]. These children typically score above the average range on intelligence quotient (IQ) tests [1]. However, giftedness is a multifaceted concept encompassing various components, and it would be reductive to define it solely based on IQ scores [2].

Piaget proposed that intelligence is not solely associated with cognitive abilities but also linked to emotional and sensory perceptions [3]. The role of intelligence in sensory processing among gifted individuals is an intriguing topic while the relationship between sensory modulation abilities and intelligence levels is known, it may take a different form in cases of heightened sensory sensitivity [4]. Individuals with high sensory sensitivity may sometimes leverage this trait to support their internal development and learning processes, whereas others may struggle to cope with such heightened sensitivity [4]. Gifted children may exhibit developmental trajectories distinct from their peers and experience hypersensitivity in areas such as touch, vision, hearing, balance, and movement during the sensory input regulation process [5]. Research indicates that gifted children often demonstrate heightened sensitivity to incoming sensory stimuli, leading to sensory discomfort, which can result in behavioral skill deficits and emotional responses [4].

Hyperacusis is a hearing disorder characterized by increased sensitivity or decreased tolerance to sounds that would not typically disturb most individuals [6]. Although data on the prevalence and characteristics of hyperacusis during childhood are limited, its prevalence in the pediatric population has been reported to range between 3.2% and 17.1% [6, 7]. Sensitive individuals may perceive environmental sounds as more stressful, leading to heightened sensitivity to auditory stimuli [8]. Everyday sounds may be perceived as unpleasant, intense, painful, and overwhelming, often seeming much louder than they actually are [6]. Research on hyperacusis is still in its early stages, and a definitive etiology or pathophysiology has not yet been medically established [9].

The literature indicates that hyperacusis is most commonly observed in children aged 3–4 years [10], and is more frequently reported in boys [11]. Autism and tinnitus are the conditions most commonly co-occurring with hyperacusis. Among children with hyperacusis, the most bothersome sounds are typically those produced by household electrical appliances. Although the literature describes a wide range of behavioral responses associated with hyperacusis, children often react by covering their ears, crying, or exhibiting aggressive behavior [8]. These behavioral responses can affect many aspects of a child’s life, including communication skills, social relationships, and academic performance. While older children may cope with hyperacusis by avoiding certain sounds or using hearing protection, younger children are less likely to have effective coping mechanisms. Consequently, parents may impose restrictions on their children’s regular activities to prevent distress, which can exacerbate social isolation. The most challenging factors for families include leaving the house, participating in social activities, and managing school performance. These challenges often lead to high levels of stress, ultimately reducing the quality of life for both the child and their family.

Studies have shown that hyperacusis is common in people with neurodevelopmental disorders that make people more sensitive to sound and emotions, like autism, giftedness, and attention-deficit/hyperactivity disorder (ADHD) [8]. Imaging studies investigating the origins of hyperacusis commonly report hyperactivity in specific brain regions among affected individuals. Current evidence suggests that hyperacusis may be associated with functional alterations in auditory and non-auditory pathways of the central nervous system, as well as heightened emotional or anxiety-based responses to sound [12, 13] Comprehensive studies have shown greater bilateral frontal and right parietal lobe activation in gifted individuals compared to healthy controls [14]. Notably, the long-distance white matter tracts are reported to be denser in gifted individuals, providing strong and efficient connections between the brain’s frontal and parietal regions [1]. This enhanced connectivity may improve their ability to process information more quickly and efficiently [1]. However, how these neurophysiological changes in the central nervous system affect sensory processing in gifted individuals remains unclear. One study found that hyperactivation of the frontal cortex and desynchronization in the gamma band are two main things that cause and make hyperacusis worse [15]. It is hypothesized that increased sensory sensitivity may lead to hyperacusis and that heightened activation and neurophysiological changes in various brain regions may also contribute to its development.

The literature has not identified any studies evaluating the presence of hyperacusis in gifted children. While research on gifted children is limited, existing studies typically focus on their social and emotional needs and the challenges they encounter. One of the unique aspects of our study is that it represents the first investigation in this domain. The aim of our study is to identify hyperacusis in gifted children and evaluate its impact on their quality of life.

## Method

The study was conducted in accordance with the Declaration of Helsinki and it was approved by Ankara Yıldırım Beyazıt University Non-Interventional Clinical Research Ethics Committee with meeting decision no:06/794 on 01.07.2024. The children and parents who participated in the study were informed of its purpose and scope and gave written informed consent.

### Participants

The study included 47 children between the ages of 7 and 12 who did not have hearing loss, were diagnosed as gifted with a score of 120 or higher on the Weschler Intelligence Scale for Children-IV (WISC-IV), and had no concomitant disabilities, and 27 age- and sex-matched children with normal intelligence. Participants were randomly selected from Rukiye Çolak Sivri Speciality Clinic.

### Research protocol

After completing the demographic information form, the families of gifted and normally intelligent children completed the ‘Parent Form of the Pediatric Hyperacusis Questionnaire (P-HQ)’ to assess hyperacusis and the ‘Quality of Life for Children (PedsQL)’ to assess quality of life.

### Pediatric hyperacusis questionnaire parent form(P-HQ)

The Pediatric Hyperacusis Questionnaire Parent Form is an 11-item assessment tool developed by Carson et al., adapted from the Khalfa Hyperacusis Questionnaire [16]. The Turkish adaptation, including its validity and reliability studies, was conducted by Öztürk-Özdeş et al. [17]. This questionnaire employs a three-point Likert-type scoring system (Yes: 2 points, Sometimes: 1 point, No: 0 points). The total score ranges from 0 to 22, with scores above 10 indicating an increased likelihood of hyperacusis. Notably, the questionnaire does not include any reverse-scored items.

### Quality of life scale for children (PedsQL)

Varni and colleagues developed a general quality of life scale to assess the physical and psychosocial experiences of children aged 2–18 years [18]. The Turkish adaptation, including its validity and reliability studies, was conducted by Çakın-Memik and colleagues [19]. The scale consists of 23 items and is scored across four subdomains. The Total Quality of Life Score (TQOLS) is calculated first, followed by subtest scores for Physical Health Total Score (PHTS), Emotional Functioning Score (EFS), Social Functioning Score (SFS), and School Functioning Score (ScFS). Responses to the questions are scored on a scale from 0 to 100. A five-point Likert-type system is used, where responses are scored as follows: “never” 100, “rarely” 75, “sometimes” 50, “often” 25, and “always” 0. The total score is obtained by dividing the sum of the responses by the number of items answered. The Quality of Life Scale for Children (PedsQL) total score is considered an indicator of how well health-related quality of life is perceived.

### Statistical analysis

Statistical analysis was performed using SPSS (statistical package for social sciences) version 26.0 software (IBM Corp.; Armonk, NY, USA). Sample size was determined using G*Power version 3.1 software (parameters: correlation of interest ρH1 = 0.5, α error rate = 0.05, power = 0.85). Histogram curves and bell curves were used to assess whether the data were normally distributed. Percentage values were used for category variables such as gender for descriptive statistics. Mean ± standard deviation values were used in the descriptive statistics of the age variable. Group 1 was composed of 47 gifted children who met the inclusion criteria, and Group 2 was composed of 27 children with normal intelligence. P-HQ and PedsQL values were compared between the groups. Student t-test or Mann-Whitney U test was used for two-group comparisons. In addition, the correlation between P-HQ, PedsQL and WISC-IV was examined. A p-value of 0.05 was considered statistically significant.

## Results

A total of 74 children participated in the study, 47 of whom were diagnosed as gifted by scoring 120 or more on the WISC-IV test between the ages of 7 and 12 (mean age 9.27 ± 1.63; 16 girls, 31 boys) and 27 children with normal intelligence (mean age 9.33 ± 1.27; 10 girls, 17 boys).

Gifted children with a WISC-IV score of 120 and above form Group 1; children with normal intelligence form Group 2 as a control group. There were no additional disabilities in the children in either group.

Comparison of the groups according to descriptive characteristics is given in Table 1. According to Table 1, the groups were similar in terms of gender and age (*p* =.877).

**Table 1:** Comparison of groups according to their descriptive characteristics

	Group 1 (*n* = 47)	Group 2 (*n* = 27)	*P*
**Age (year)**			
Mean ± SD (Min-Max)	9.27 ± 1.63 (7–12)	9.33 ± 1.27 (7–12)	0.877
**Gender**			
Female (n) Male (n)	%34 (16) %66(31)	%37 (10) %63 (17)	

**Table 2:** Comparison of PedsQL between groups

	Group 1 X ± SD (Min.-Max.)	Group 2 X ± SD (Min.-Max.)	*P*	%95 CI
				[Lower/Upper]
PHTS	65.02 ± 19.21 (31.25–100)	81.36 ± 14.30 (46.88–100.00)	0.000**	[-24.81/-7.86]
EFS	58.08 ± 23.55 (30.00- 100)	70.00 ± 20.75 (30.00- 100)	0.032*	[-22.78/-1.04]
SFS	70.63 ± 21.45 (10.00-100.00)	84.44 ± 18.82 (20.00-100.00)	0.007**	[-23.69/-3.91]
ScFS	(5.00-100.00)	(10.00-100.00)	.000^aa^	[-24.98/-5.96]
TQOLS	258.78 ± 63.90 (119.38-391.88)	316.92 ± 56.77 (151.88–390.00)	0.000**	[-87.70/-28.56]

Data were presented as mean ± standard deviation and CI (confidance interval). PedsQL consists of 23 items– that are grouped in 4 subscales or domains; TQOLS: Total Quality of Life Score; PHTS: Physical Health Total Score EFS: Emotional Functioning Scale, SFS: Social Functioning Scale ve ScFS: School Functioning Scale. Within- group changes were presented as * *p* <.05 and ** *p* <.01. X ± SD: Mean ± Standart Deviation, Paired samples T Test *p < 0,05, Mann Whitney U Test ^a^*p*<0,05 ^aa^*p*<0,001

When the quality of life scores of the children in Group 1 and Group 2 were compared, a significant difference was found in all sub-parameters of the test and in the Total Quality of Life Score; (TQOLS) (*p* <.05). Group 1, which consisted of gifted children, had worse scores on the Physical Health Total Score (PHTS) (*p* =.000), the Emotional Functioning Scale (EFS) (*p* =.032), the Social Functioning Scale (SFS) (*p* =.007) and the School Functioning Scale (ScFS) (*p* =.000) (Table 2).

When the Pediatric Hyperacusis Questionnaire Parent Form scores of the groups were compared, a statistically significant difference was found (*p* =.000) (Table 3). The hyperacusis scores of group 1 were worse than those of the control group.

**Table 3:** Comparison of P-HQ between groups

	Group 1 X ± SD (Min.-Max.)	Group 2 X ± SD (Min.-Max.)	*P*	%95 CI
				[Lower/Upper]
P-HQ	10.40 ± 4.62 (0.00–19.00)	5.77 ± 5.17 (0.00–20.00)	.000**	[2.29/6.95]

Data were presented as mean ± standard deviation and CI (confidance interval). P-HQ: Pediatric Hyperacusis Questionnaire Parent Form. Within-group changes were presented as * *p* <.05 and ** *p* <.01. X ± SD: Mean ± Standart Deviation, Paired samples T Test *p <.05

**Table 4:** Distribution of hyperacusis in the gifted group according to gender

Gender	With Hyperacusis (*n*)	With Hyperacusis (*n*)	Total(*n*)
Male	20	61.7%	31
Female	6	37.5%	16
Total	**26**	**53.2%**	**47**
p		0.049*	

Chi-square test; within- group changes were presented as * *p* <.05

Chi-square test was used to evaluate the relationship between gender and hyperacusis. Hyperacusis rate was calculated as 61.7% in males and 37.5% in females. As a result of the analysis, there is a statistically significant relationship between gender and hyperacusis (χ² [1] = 0.3888, *p* =.049) (Table 4). This result shows that the prevalence of hyperacusis is higher in males.

Correlation analyses were performed between the children’s quality of life scores and hyperacusis scores. Accordingly, there are strong, statistically significant negative correlations between P-HQ and TQOLS (*r*=-.556) and its sub parameters PHTS (*r*=-.407), EFS (*r*=-.490), SFS (*r*=-.367), and ScFS (*r*=-.529). According to the hyperacusis scoring guide, there is a negative correlation between the quality-of-life score because a higher hyperacusis score indicates poorer performance (Table 5).

There were strong, statistically significant positive correlations between the P-HQ and WISC-IV intelligence test scores (*r* =.434). As individuals’ intelligence test scores increased, their hyperacusis scores also increased (Table 5).

**Table 5:** Correlation between PedsQL, P-HQ and WISC-IV

		PHTS	EFS	SFS	ScFS	TQOLS	*P*-HQ	WISC-IV
PHTS	r	1	.485**	.622**	.366**	0.759**	− 0.407**	− 0.349**
	p		0.000	0.000	0.001	0.000	0.000	0.002
EFS	r	.485**	1	0.481**	.553**	.812**	− 0.490**	− 0.173
	p	0.000		0.000	0.000	0.000	0.000	0.141
SFS	r	0.622**	0.481**	1	0.492**	0.806**	− 0.367**	− 0.190
	p	0.000	0.000		0.000	0.000	0.001	0.104
ScFS	r	0.366**	0.553**	0.492**	1	0.766**	− 0.519**	− 0.225
	p	0.001	0.000	0.000		0.000	0.000	0.054
TQOLS	r	0.759**	0.812**	0.806**	0.766**	1	− 0.556**	− 0.304**
	p	0.000	0.000	0.000	0.000		**0.000**	**0.008**
P-HQ	r	− 0.407**	− 0.490**	− 0.367**	− 0.519**	− 0.556**	1	.434**
	p	0.000	0.000	0.001	0.000	0.000		0.000
WISC-IV	r	− 0.349**	− 0.173	− 0.190	− 0.225	− 0.304**	0.434**	1
	p	0.002	0.141	0.104	0.054	0.008	0.000	

Spearman’s/Pearson’s correlation; *correlation is significant at the 0.05 level, **correlation is significant at the 0.01 level. Bolded areas indicate that the statistical analysis is significantly different

## Discussion

In neurodevelopmental disorders such as autism, ADHD, and giftedness, altered reactivity due to sensory sensitivity—either increased or decreased—is commonly observed [12, 13]. This altered reactivity may involve tactile, olfactory, gustatory, visual, vestibular, or auditory modalities. Our study evaluated hyperacusis, defined as increased auditory reactivity, and its impact on the quality of life in gifted children. While there have been studies of hyperacusis in autism and ADHD that are available, this research represents the first study focusing on gifted children. A study assessing the prevalence of hyperacusis in children diagnosed with ADHD found that 36.7% exhibited it. These children were predominantly male and displayed responses such as covering their ears, avoiding sounds, and experiencing pain in the ears during sound exposure [13]. Similarly, the prevalence of hyperacusis in children with autism spectrum disorders has been reported to range from 18 to 69%, with a higher occurrence in males [20]. Consistent with these findings, our study observed that most of the gifted participants were male, and 61.7% reported hyperacusis-related complaints, indicating a notably high prevalence (χ² [1] = 0.3888, *p* =.049).

Gifted children’s altered sensory sensitivities, whether heightened or diminished, can lead to limitations in coordination skills, motor abilities, and daily life skills [4]. These limitations often result in reduced social skills, low self-esteem, negative self-perception, and behavioral problems [4, 5]. Due to these traits, children may face negative judgments from their surroundings, including peers, teachers, parents, and relatives. It is known that such sensory sensitivities and associated challenges are linked to the child’s level of intelligence [21]. Gere et al. reported that as individuals’ intelligence levels increased, so did their sensitivities and behavioral problems [5]. Supporting the literature, our study revealed a positive correlation between intelligence level and hyperacusis (*p* =.000, *r* =.434**). Moreover, we demonstrated that gifted children reported significantly more intense hyperacusis complaints compared to children of the same age group with average intelligence. This finding suggests that gifted children may be more sensitive to sounds and vibrations, and their brains may be working more actively in some areas. These changes may also play a role in the development of hyperacusis.

The challenges faced by gifted children due to their sensory sensitivities predominantly affect their social communication skills with peers, emotional processes, and academic performance [5, 22]. These children often struggle to integrate during peer play, fail to succeed in sports activities, and avoid crowded environments. When forced into such situations, they may experience crying spells or anger outbursts, leading to their alienation [5]. While older children may develop coping mechanisms to manage their changing sensory sensitivities, younger children find it particularly difficult to deal with this sensory and emotional burden. When families fail to understand the reasons fully behind these challenges, they often struggle to manage the situation, which increases their stress levels and leads to imposing restrictions on the child’s daily life. This, in turn, negatively impacts the parent-child relationship and the quality of life for both parties. In the same way, this study found that gifted children scored lower on the physical functioning, sensory, social, and school domains of the Pediatric Quality of Life Inventory (PedsQL), as well as the overall score, compared to their averagely smart peers. In relation to this, we observed a negative correlation between intelligence level and quality of life, particularly in the physical functioning subtest. This finding supports the notion that as intelligence levels increase, physical functioning and overall quality of life tend to decrease. Additionally, our study demonstrated a negative correlation between hyperacusis and quality of life. Higher levels of hyperacusis were associated with reduced scores in physical functioning, sensory, social, and school domains, as well as overall quality of life. These findings are critical in shaping our approach to gifted children, highlighting the need for targeted strategies to address their unique challenges.

Although there is no “gold standard” method for assessing hyperacusis, self-report scales, frequency-specific measurements of loudness discomfort levels, and psychoacoustic tests are commonly used in adults [17, 23]. However, such assessment tools are limited for children. One possible reason for this gap is that the underlying mechanisms of hyperacusis remain largely unknown [9]. Several physiological and psychological processes have been suggested as possible causes, but researchers are still not sure if decreased sound tolerance is linked to problems in the auditory brainstem, problems with low-level auditory processing, unusually strong emotional responses to certain auditory stimuli, or a mix of these factors [24]. We believe that gifted children with complaints of hyperacusis should undergo audiological evaluations in addition to completing self-report questionnaires and family interviews. These evaluations should assess the functions of both cochlear and retrocochlear structures to gain a more comprehensive understanding of their auditory processing.

Given the recent emergence of hyperacusis as a topic of interest and the limited evidence concerning children, professionals such as physicians, psychologists, audiologists, educators, and other specialists are often insufficiently prepared to support families dealing with this condition. There is no treatment specifically developed for children with hyperacusis, nor is there empirical evidence supporting the efficacy of existing treatments for this population [8]. Additionally, documented treatment approaches and their combinations (e.g., pharmacological interventions with psychological therapy or sound therapy) show significant variability [8]. However, none of these approaches provide detailed definitions of their components, nor do they clearly report patient outcomes. Implementing appropriate assessment and treatment strategies can aid in identifying and supporting these children both in society and at school. The children and their families can improve their quality of life by addressing their challenges.

Professionals working with gifted children should carefully consider these children’s changing sensory sensitivities during evaluations. Understanding sensory sensitivities and reactions is crucial for managing processes in education, therapy planning, and family counseling through a multidisciplinary approach. Our study is limited by the small sample size and the recruitment of participants from a single private clinic. To enhance the generalizability of our findings, future research should involve a larger sample in a multicenter study.Although the test results were positive, definitive conclusions about the causal relationship between hyperacusis and quality of life or IQ cannot be drawn based solely on correlations. A more rigorous, objective methodological approach and further research are necessary to explore this connection. We recommend that future research focus on improving the assessment of hyperacusis in children through objective measures, such as loudness discomfort levels and otoacoustic emissions (OAE) suppression. Additionally, studies should provide clinical guidance on managing children’s difficulties and identifying necessary community adjustments.

## Conclusion

Our findings indicate that a significant proportion of gifted children experience hyperacusis, with the severity of complaints increasing alongside intelligence levels. We believe that hyperacusis and its impact on quality of life should not be overlooked in gifted children. Whenever possible, gifted children should be assessed for hyperacusis and sensory sensitivities. We advocate for the development of individualized therapy plans to address the challenges arising from sensory sensitivities that affect their quality of life. Our results highlight the necessity of incorporating hyperacusis assessment and therapy into practices involving gifted individuals.

## Data Availability

The datasets generated during and/or analyzed during the current study are available on reasonable request.
